# Novel insights into in‐vivo diffusion tensor cardiovascular magnetic resonance using computational modelling and a histology‐based virtual microstructure

**DOI:** 10.1002/mrm.27561

**Published:** 2018-10-23

**Authors:** Jan N. Rose, Sonia Nielles‐Vallespin, Pedro F. Ferreira, David N. Firmin, Andrew D. Scott, Denis J. Doorly

**Affiliations:** ^1^ Department of Aeronautics Imperial College London London United Kingdom; ^2^ Cardiovascular Magnetic Resonance Unit The Royal Brompton Hospital London United Kingdom; ^3^ National Heart and Lung Institute Imperial College London London United Kingdom

**Keywords:** DT‐CMR, Histology, Microstructure, Monte Carlo, Sheetlets, Simulations

## Abstract

**Purpose:**

To develop histology‐informed simulations of diffusion tensor cardiovascular magnetic resonance (DT‐CMR) for typical in‐vivo pulse sequences and determine their sensitivity to changes in extra‐cellular space (ECS) and other microstructural parameters.

**Methods:**

We synthesised the DT‐CMR signal from Monte Carlo random walk simulations. The virtual tissue was based on porcine histology. The cells were thickened and then shrunk to modify ECS. We also created idealised geometries using cuboids in regular arrangement, matching the extra‐cellular volume fraction (ECV) of 16–40%. The simulated voxel size was 2.8 × 2.8 × 8.0 mm^3^ for pulse sequences covering short and long diffusion times: Stejskal–Tanner pulsed‐gradient spin echo, second‐order motion‐compensated spin echo, and stimulated echo acquisition mode (STEAM), with clinically available gradient strengths.

**Results:**

The primary diffusion tensor eigenvalue increases linearly with ECV at a similar rate for all simulated geometries. Mean diffusivity (MD) varies linearly, too, but is higher for the substrates with more uniformly distributed ECS. Fractional anisotropy (FA) for the histology‐based geometry is higher than the idealised geometry with low sensitivity to ECV, except for the long mixing time of the STEAM sequence. Varying the intra‐cellular diffusivity (*D*_IC_) results in large changes of MD and FA. Varying extra‐cellular diffusivity or using stronger gradients has minor effects on FA. Uncertainties of the primary eigenvector orientation are reduced using STEAM.

**Conclusions:**

We found that the distribution of ECS has a measurable impact on DT‐CMR parameters. The observed sensitivity of MD and FA to ECV and *D*_IC_ has potentially interesting applications for interpreting in‐vivo DT‐CMR parameters.

## 1 Introduction

Diffusion tensor imaging (DTI) is unique in enabling a non‐invasive inference of the underlying tissue microstructure.^1^ While historically the DTI literature has focussed on the brain,^2^ recent developments in diffusion tensor cardiovascular magnetic resonance (DT‐CMR) have overcome the difficulties associated with measuring diffusion in the dynamic environment of the heart.^3^−8 This has facilitated in‐vivo assessment of microstructural abnormalities in a number of patient cohorts.^9^, ^10^, ^11^, ^12^

The nature of a beating heart means that traditional Stejskal–Tanner‐type diffusion‐weighted spin echo techniques cannot be used. Several alternatives have been proposed to cope with the effects of cardiac motion during the acquisition. These include stimulated echo acquisition mode,^3^, ^4^, ^5^ which applies short gradients at the same time in two successive cardiac cycles; and motion‐compensated spin echo imaging,^7^, ^8^, ^13^ where the diffusion‐encoding gradient design eliminates the effect of constant velocity and acceleration.

In the myocardium, the primary eigenvector of the diffusion tensor (${\vec{E}}_{1}$) aligns with the cardiomyocyte long axis^14^ whose orientation varies linearly from epi‐ to endocardium^15^ and is described by the helix angle (HA).^16^ The cardiomyocytes are also grouped in aggregates known as sheetlets, separated by collagen‐lined shear layers,^16^, ^17^ and recent validation work has shown that the secondary eigenvector of the diffusion tensor (${\vec{E}}_{2}$) lies within the sheetlet plane.^18^ For healthy subjects, E2A (used to describe ${\vec{E}}_{2}$ orientation) varies as the heart contracts.^19^ However, studies in hypertrophic and dilated cardiomyopathy patients^18^, ^20^, ^21^ have demonstrated abnormalities in the re‐orientation of sheetlets. There are also pathological changes in other diffusion tensor parameters such as fractional anisotropy (FA) and mean diffusivity (MD),^9^, ^10^, ^12^ but these could be the consequence of a number of underlying changes in microstructure.

The exact relationship between measured DT‐CMR signal and the underlying microstructure is not currently well understood. This is further complicated by the fact that techniques typically used in vivo have intrinsically low signal‐to‐noise ratios and consequently voxel sizes are large (ca. 3 × 3 × 8 mm^3^).^5^ During typical measurements, water diffuses tens of micrometres and thereby probes the local tissue structure. However, the size of the imaging voxel has an averaging effect on the microstructural information provided. A heterogeneous microstructure may hide geometric features localised only to sub‐voxel‐sized regions.^22^ In addition, the range of cardiomyocyte orientations, multiple shear layer orientations, and non‐uniform cardiomyocyte size distributions found within a voxel contribute to the uncertainty. As a result it is difficult to predict exactly how local microstructural abnormalities manifest themselves in the DT‐CMR results.

Computational simulations are an increasingly common method used to investigate hindered and restricted diffusion in DTI. This allows a unique opportunity to investigate physiological changes such as cardiomyocyte hypertrophy or disarray in a controlled in‐silico environment. Confounding effects can be studied and simulation results may identify the underlying changes in microstructure that cause changes in DT‐CMR results. It also allows for assessment of the sensitivity of the different sequences typically used in vivo.

Both continuum solutions of the Bloch–Torrey Equations^23^ and Monte Carlo random walk methods, which are significantly more efficient for complex geometries, can be used to synthesise DT‐CMR data in a known microstructure. Previous studies have reported DTI simulations in a number of organs,^24^, ^25^, ^26^, ^27^, ^28^, ^29^, ^30^ often modelling geometries using ordered or random arrays of cuboids or cylinders. These investigations lack the long mixing times and large voxels that are typical for DT‐CMR and the simplified shapes may not be adequate to represent the complicated architecture of cardiac muscle tissues.

The aim of this present work is to perform Monte Carlo random walk simulations with a realistic histology‐based geometry, using non‐analytical arbitrarily shaped virtual cardiomyocytes, in order to minimise potential systematic errors caused by idealised geometries. We present a method to compensate for fixation‐induced tissue shrinkage when constructing the simulation substrate from histology and study how the proportion and distribution of extra‐cellular space (ECS) within sheetlets and the amount of ECS, quantified through the extra‐cellular volume fraction (ECV), affects DT‐CMR results. Simulations are performed for various in‐vivo DT‐CMR pulse sequences, covering both long and short mixing times for regular and high‐performance gradient systems, with a realistic voxel size. By varying the intra‐ and extra‐cellular diffusivity—model parameters that may correspond to physiological changes in the tissue—we show the sensitivity of the sequences to properties intrinsic to the tissue and eigenvector uncertainties.

## 2 Methods

### 2.1 Histology‐based simulation substrate

**Figure 1:**
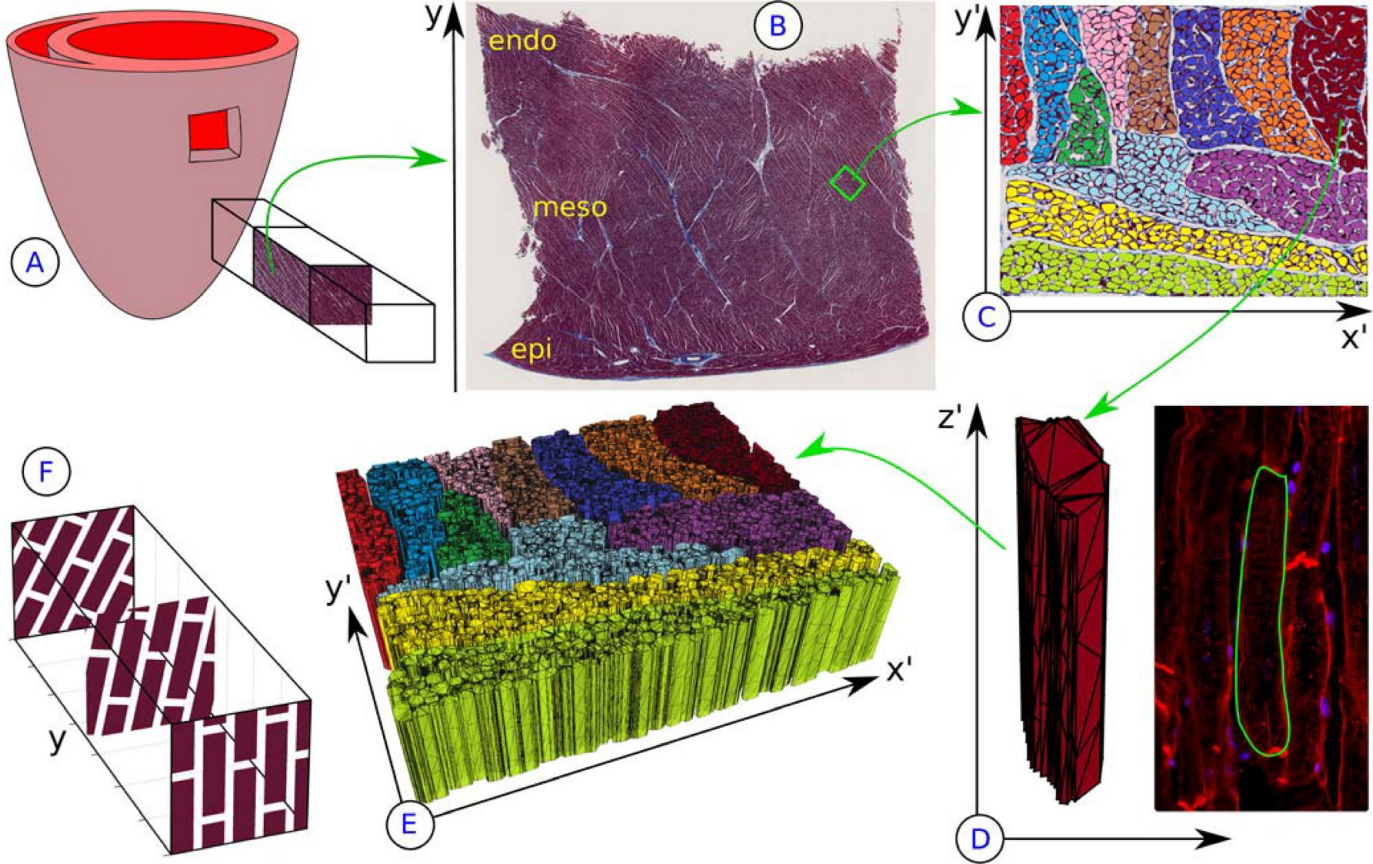
A transmural block was cut from the mid‐level left ventricle of a 30 kg Yorkshire swine (A). Transmural–longitudinal sections were imaged (B) and a region of interest (ROI) from the mesocardium selected and manually segmented (C). Each cardiomyocyte is extruded along the image‐normal and meshed (D). Size and shapes may be compared with confocal microscopy. The resulting 3D block of virtual cells (E) is then replicated to fill an entire voxel by stacking and applying a 10^∘^/mm rotation

The virtual microstructure is based on pig heart histology^18^ (Masson's trichrome stain, 20× magnification). Transmural–longitudinal 10 μm thick sections were cut from the mid‐level left ventricle, see Figure 1A. The heart was obtained from a 30 kg Yorkshire swine, arrested in a contracted (systolic‐like) state using barium chloride and with ventricular mass of 88 g.

Figure 1B shows the location of the region of interest (ROI) with dimensions 500 × 400 μm, chosen as representative tissue of the surrounding mesocardium. Such a region shows myocytes cut perpendicular to their long axis, thereby reducing errors in the cross‐section that may otherwise arise from an angled section.

In total, 1182 cardiomyocytes in 12 sheetlets were manually segmentated, see Figure 1C. All subsequent processing operations were performed in MATLAB (R2017b, The MathWorks, Inc., Natick, Massachusetts). The pixel boundaries of the cell cross‐sections were simplified as polygons with an average of 99 vertices (range: 14–320) and cell membranes reduced to have zero thickness. These objects were then extruded in their local *z* ^′^‐direction normal to the slice to create 3D polyhedrons (Figure 1D), with random uniformly distributed lengths in the range 114–126μm.^31^, ^32^, ^33^

The simulation substrate is constructed by replicating this “building block” (Figure 1E). For a given transmural position *y* in the voxel, the blocks with local coordinate system *x* ^′^ *z* ^′^ are tiled as shown in Figure 1F with every other row in *z*′ (direction of the long axis of cardiomyocytes) shifted by half a block to eliminate long straight channels in the *z*′‐direction. A sinusoidal *z* ^′^‐displacement is applied to the myocyte vertices to mitigate channels along the *x* ^′^‐direction. This is repeated along the transmural *y*‐direction by applying a rotation of 10^∘^/mm (4^∘^/block),^34^, ^35^, ^36^ corresponding to a 12 mm thick wall and a HA range of +60–−60^∘^.

### 2.2 Random walk algorithm

The self‐diffusion of H_2_O molecules can be modelled as a Monte Carlo random walk of massless particles. By ignoring collisions this is parallelised such that each particle is treated as an independent random walker. The algorithm outlined below is described for a single particle, repeated *N*_P_ times with a different random number stream to simulate *N*_P_ particles (chosen in Supporting Information Figure S1). A uniformly distributed random initial position is chosen in a cuboid larger than the simulated voxel by $\pm \sqrt{6DT}$ (with *D* the largest diffusivity and *T* the total simulation time) to mitigate the effect of particles leaving and entering the voxel throughout the simulation. After each particle has completed the pulse sequence simulation, its signal is combined with that of all other particles to compute the diffusion‐weighted MRI signal.

At the beginning of each timestep of duration d*t*, an initial displacement vector ${\vec{R}}_{0}$ is drawn with each of its components *R*_*i*_ following a normal distribution with mean *μ* = 0 and variance σ^2^ = 2*D*d*t*. The step length is therefore scaled by the local diffusivity *D*, which is either the intra‐ or extra‐cellular diffusivity (*D*_IC_, *D*_EC_). To ensure robustness in the random walk and limit the search space of possible myocyte boundary intersections, the normal distribution is limited to ±5σ by rejection sampling (see the convergence study in Supporting Information Figure S2). Values of *R*_*i*_ larger than this are discarded and a new random value is drawn.

Depending on the local tissue geometry (represented as a triangulated surface mesh) encountered in the particle's path, sub‐steps ${\vec{R}}_{j}$ are performed such that $\sum_{j}^{M}\|{\vec{R}}_{j}\left\|\;=\;\right\|{\vec{R}}_{0}\|$, where *M* is one more than the number of boundary intersections encountered in the current timestep. The time‐varying position $\vec{r}\left(t\right)$ of the particle after performing a single timestep d*t* is then given by

(1) $$\vec{r}\left(t+dt\right)\;=\;\vec{r}\left(t\right)+\sum_{j}^{M}{\vec{R}}_{j}.$$

The Möller–Trumbore algorithm,^37^ accelerated through bounding boxes, was used to test for intersections with cardiomyocyte boundaries. Elastic reflection was modelled using the local boundary normal $\vec{n}$ of the intersected mesh surface, where

(2) $${\vec{R}}_{\text{new}}\;=\;{\vec{R}}_{\text{old}}-2\left({\vec{R}}_{\text{old}}\cdot \vec{n}\right)\vec{n}.$$

**Figure 2:**
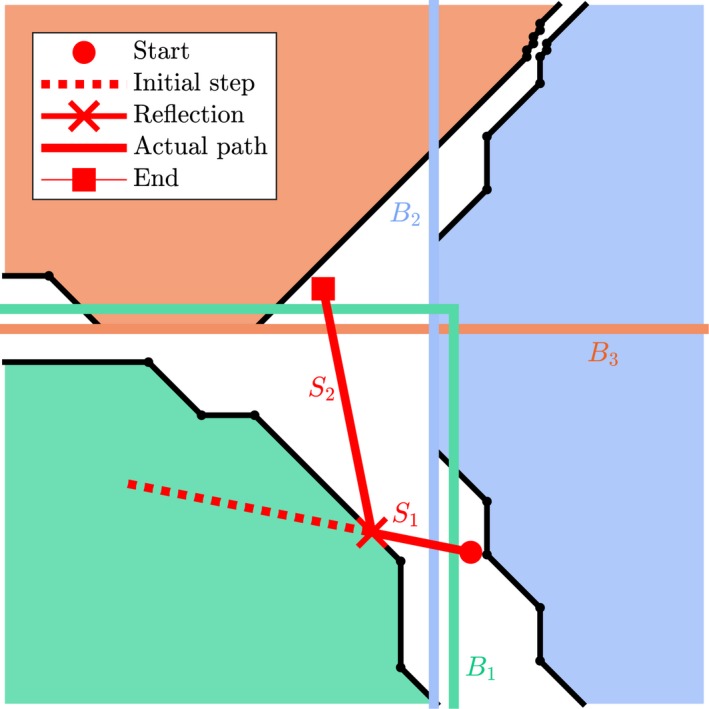
2D example of one extra‐cellular particle performing a single random walk time step of length $\|{\vec{R}}_{0}\|\;=\;3\;\mu \text{m}$. The dashed line indicate the initial step vector before sub‐step *S*_1_ and the solid lines represent the actual path taken by the particle after resolving all barrier interactions. During *S*_1_, bounding boxes *B*_1_ and *B*_2_ are considered, whereas during *S*_2_ cell barriers belonging to *B*_1_ and *B*_3_ are affected

The remaining step size after the reflection is used as the initial step size in the next sub‐step. Figure 2 illustrates how a possible displacement is resolved.

Simulations were performed using MATLAB (R2017b) with the Parallel Computing Toolbox for parallelisation of the random walk. Computationally expensive tasks (bounding box and ray–triangle intersection tests) were implemented in C. Parameter studies used the Imperial College Research Computing Service facilities,^38^ utilising up to 50 compute nodes each with two Intel Xeon E5‐2660v2 processors (10 cores at 2.2 GHz clock speed each) and 128 GB of RAM.

### 2.3 MRI data synthesis

**Figure 3:**
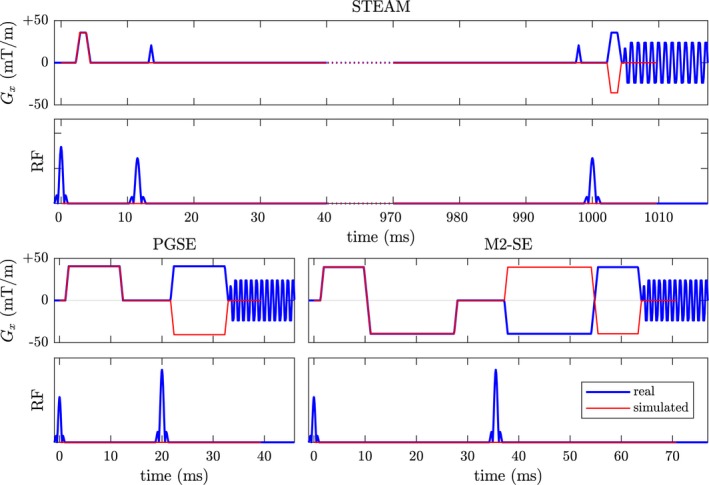
Comparison of real and simulated pulse sequences used for simulations. The simplified sequence starts at the peak of the RF pulse and ends at the echo. The 180^∘^ pulse (spin echo) and the two 90^∘^ pulses (STEAM) are considered by reversing the polarity of the second gradient. Readout and refocusing gradients are not simulated

We simulated an imaging voxel of size 2.8 × 2.8 × 8.0 mm^3^. Three pulse sequences were considered: Stejskal–Tanner pulsed‐gradient spin echo (PGSE),^39^ second‐order motion‐compensated spin echo (MCSE),^7^ and monopolar simulated echo acquisition mode (STEAM).^4^ Simulations were based on simplified versions of these three sequences as shown in Figure 3. A *b*‐value of 450 m/ms^2^ with reference value of 0 was used. To investigate the effect of *G*_max_ we performed all experiments with maximum gradient strengths of 40 and 80 mT/m per axis. The pulse sequence parameters are listed in Table 1 and their definitions are shown in in Supporting Information Figure S4. Each simulated sequence starts with *t* = 0 at the initial 90^∘^ RF pulse and ends at the echo (*t* = TE for spin echo, *t* = TE + TM for stimulated echo). The slice‐select and readout gradients are not simulated and an idealised instantaneous acquisition is assumed. Trapezoidal diffusion gradients are applied on two scanner axes simultaneously, creating a total of six diffusion‐encoding gradients, as frequently used for in‐vivo studies.^9^, ^40^, ^41^, ^42^ RF pulses were idealised as instantaneous and perfect 90 or 180^∘^ pulses. To account for the 180^∘^ pulse or pair of 90^∘^ pulses, the polarity of the second half of the diffusion encoding is reversed.

**Table 1:** Characteristic parameters of the simulated DT‐CMR pulse sequences for two values of maximum gradient strength (*G*_max_)

Parameter	PGSE	M2‐SE	STEAM			
*G*_max_ (mT/m)	40.572	80	39.478	80	35.690	80
Δ (ms)	20.547	16.976	n/a	n/a	1000	1000
TE (ms)	39.332	31.529	70.757	53.709	23.824	22.226
TM (ms)	n/a	n/a	n/a	n/a	988.088	988.887
*ɛ* (ms)	0.676	1.333	0.661	1.333	0.595	0.667
*δ* (ms)	9.921	4.385	n/a	n/a	0.977	0.034
*δ*_1_ (ms)	n/a	n/a	7.819	5.430	n/a	n/a
*δ*_2_ (ms)	n/a	n/a	16.299	7.997	n/a	n/a

Notes:

The *δ* values are gradient flat‐top durations and *ɛ* is the ramp‐up/ramp‐down time.

The pulse sequences are discretised into *N*_T_ timesteps. To ensure robustness in the random walk algorithm and accuracy in representing the gradients, maximum timesteps of d*t*_max,free_ = 1 ms and d*t*_max,grad_ = 0.1 ms are enforced. As each particle *i* performs the random walk, it acquires phase according to

(3) $$\phi_{i}\;=\;\gamma \int_{0}^{T}\vec{G}\left(t\right)\cdot {\vec{r}}_{i}\left(t\right)dt$$

where $\vec{G}\left(t\right)$ is the time‐dependent gradient vector.

At the end of the random walk, only particles remaining in the voxel are processed. The signal attenuation *A* obtained at the end of the simulation for all *i* = 1…*P* particles with position $\vec{r}$ inside the voxel Ω is described by

(4) $$A\;=\;\frac{1}{P}∥\sum_{\left\{\forall i:{\vec{r}}_{i}\in \Omega \right\}}e^{-I\phi_{i}}∥,$$

where $I\;=\;\sqrt{-1}$. The diffusion tensor and DT‐CMR parameters are then calculated using an unweighted linear inversion.^43^ We obtained the eigenvectors (${\vec{E}}_{1}$, ${\vec{E}}_{2}$, ${\vec{E}}_{3}$), eigenvalues (λ_1_, λ_2_, λ_3_), MD, FA, and tensor mode.^44^ For scalar quantities, we report the median and bounds of the two‐sided asymmetric 95% confidence interval.^45^ For the eigenvectors, we calculated the cone of uncertainty following^46^ and report the one‐sided 95% confidence interval of eigenvector uncertainties d*E*_1_, d*E*_2_, and d*E*_3_.

Based on a convergence study described in in Supporting Information Figure S1, simulations were performed with 10 realisations of each experiment using *N*_P_ = 10^4^ and *N*_T_ = 10^3^.

### 2.4 Distribution of extra‐cellular space

To study the effect of ECV and the distribution of ECS, we morphed the initial 2D segmentation to grow or shrink the cardiomyocytes by modifying their inner diameter. First, the manually segmented cardiomyocyte cross‐sections were progressively thickened by adding pixels to the boundary of each cell while enforcing sheetlet boundaries to prevent myocytes from growing into the shear layers and disrupting the overall tissue structure. Once thickened to the maximum possible degree, i.e. a one‐pixel gap between cells within a sheetlet, myocytes were gradually shrunk to uniformly grow the ECS. In total, 10 ECV values between 16 and 41% were used as simulation substrates.

Additionally, a building block was created consisting only of uniform cuboids which match the mean cardiomyocyte cross‐sectional area. The spacing between cuboids was adjusted to match the corresponding ECV of the histology‐based substrate.

### 2.5 Values for diffusivity

Both the magnitudes of *D*_IC_ and *D*_EC_ as well as their ratio affect the DT‐CMR results. We performed a parameter study on a histology‐based substrate with ECV = 25% and used two values of *D*_EC_: 2.0 and 3.0 μm^2^/ms. The intra‐cellular diffusivity was varied in steps of 0.5 μm^2^/ms, from 1.0 μm^2^/ms to the value of *D*_EC_.

The interaction of ECV and diffusivity was investigated by performing the ECV simulations with both *D*_IC_ = 1.5 μm^2^/ms, *D*_EC_ = 3.0μm^2^/ms (the diffusion of water at 37^∘^C^47^) and *D*_IC_ = 1.0 μm^2^/ms, *D*_EC_ = 2.0 μm^2^/ms, which includes a reduced diffusivity to account for extra‐cellular structures that impede free diffusion.

## 3 Results

### 3.1 Virtual tissue

**Figure 4:**
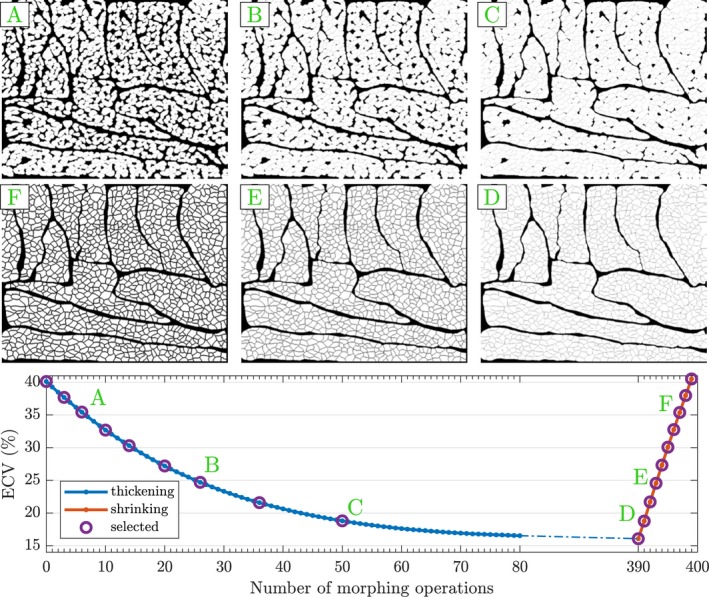
Change in extra‐cellular volume fraction as a result of progressively performing cell morphing operations. Images A–F (clockwise from top left) show cardiomyocyte cross‐sections as the cells are thickened (A–C) until reaching ECV_min_ and then shrunk (D–F). Each column shows cross‐sections with equal extra‐cellular volume fraction (ECV) but different distribution of extra‐cellular space (ECS). The circled points correspond to the selected simulation substrates used to study the effect of ECV

Figure 4 shows how ECV varies during morphing of the cardiomyocyte geometry. Starting with ECV = 40%, the thickening of myocytes reduces ECV. The smallest inter‐cellular gaps close first, leaving larger isolated gaps within the sheetlets. In total 390 thickening operations are needed to reduce ECV to ECV_min_ = 16%. However, only 9 iterations are necessary to return to an ECV of 40% when shrinking the cells.

### 3.2 Effect of diffusivity

**Figure 5:**
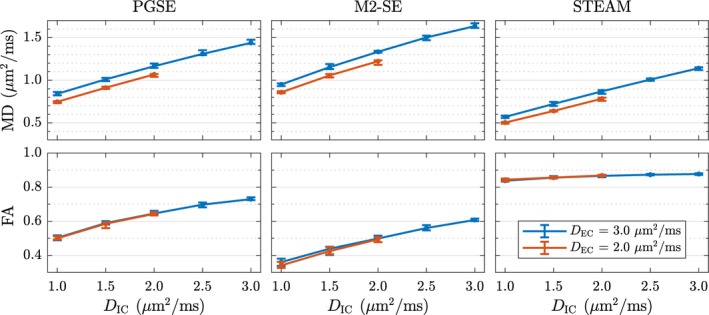
Varying both intra‐ and extra‐cellular diffusivity (*D*_IC_, *D*_EC_) reveals the sensitivity of DT‐CMR parameters to both the ratio and magnitudes of prescribed compartment‐specific free diffusivities. Each data point is the median value obtained from 10 realisations and the error bars indicate the two‐sided 95% confidence interval.

The choice of compartment‐specific diffusivity values has a strong impact on the results. Figure 5 shows the sensitivity of MD and FA to *D*_IC_ for two different values of *D*_EC_. MD increases almost linearly with changes in *D*_IC_. For *D*_EC_ = 3.0 μm^2^/ms the slopes of linear fit *m* are 0.301/0.344/0.285(μm^2^/ms)/% for PGSE/M2‐SE/STEAM respectively and *R* ^2^ > 0.994. The slopes and coefficients of determination for *D*_EC_ = 2.0 μm^2^/ms are comparable, but there is a constant offset of approximately −0.1 μm^2^/ms.

FA appears unaffected by *D*_EC_, but is dependent on *D*_IC_. This relationship is visibly not linear, but FA monotonically increases with *D*_IC_. Here, STEAM shows less of a dependence than PGSE or M2‐SE.

The sensitivity of MD to *D*_IC_ is approximately constant for all three sequences, with values for MD increasing by 0.59/0.68/0.57 μm^2^/ms (PGSE/M2‐SE/STEAM) for an increase of *D*_IC_ = 1.0 to 3.0 μm^2^/ms. All available data is presented in Supporting Information Figures S5 and S6.

### 3.3 Sensitivity to prescribed extra‐cellular volume fraction

**Figure 6:**
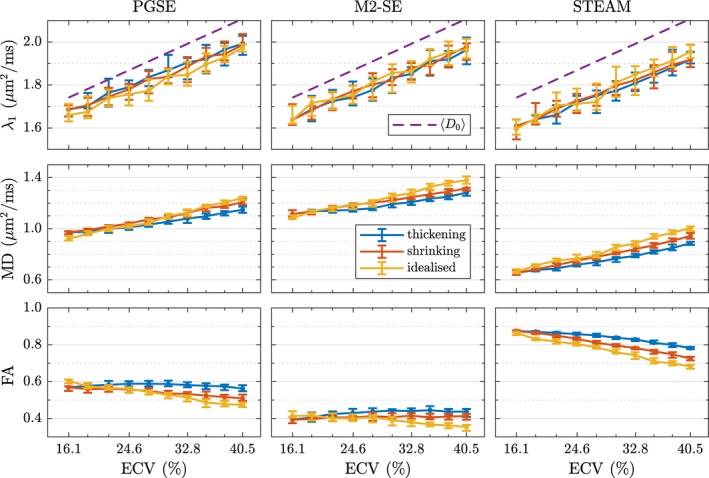
The change in diffusion tensor parameters is plotted as a function of changing the extra‐cellular volume fraction (ECV) by morphing the simulation substrate. Three different geometry types are considered here: A histology‐based geometry where cells were morphed by thickening to decrease ECV from 40%, a histology‐based geometry where cells were morphed by shrinking to increase ECV from 16%, and an idealised geometry consisting of cuboids. Each data point is the median value obtained from 10 realisations and the error bars indicate the two‐sided 95% confidence interval.

There are differences in how the DT‐CMR parameters in Figure 6 vary with ECV, depending on the simulation substrate and type of pulse sequence used.

For the idealised cuboid geometry, λ_1_ and MD both increase linearly with ECV at a rate varying from 0.012 to 0.014(μm^2^/ms)/% (compare volume‐weighted $m_{\left\langle D_{0}\right\rangle }\;=\;0.015(\mu {\text{m}}^{2}$/ms)/%) and *R* ^2^ ranging from 0.988 to 0.993 for all three sequences. FA decreases linearly with ECV: *R* ^2^ > 0.948.

Both the thickened and shrunk histology‐based geometries share the same behaviour for λ_1_. They are linear with high *R* ^2^ values but have different slopes for MD: *m* = 0.0074/0.0064/0.0092(μm^2^/ms)/% for thickening, and *m* = 0.0099/0.0080/0.0116(μm^2^/ms)/% for shrinking. Overall, the results show that modifying the ECS by shrinking cells or thickening cells to the same ECV yields different MD values. For a given ECV, we observe a higher MD and lower FA when shrinking compared to thickening. Specifically, when starting from the initial segmentation, the thickening (growing) of myocytes reduces the MD observed by PGSE/M2‐SE/STEAM from 1.15/1.28/0.89 μm^2^/ms at 40% ECV to 0.97/1.12/0.66 μm^2^/ms at 16% ECV, respectively. Subsequent progressive shrinking of the cells from ECV_min_ = 16 to 41% increases MD to 1.21/1.31/0.94 μm^2^/ms.

For the long mixing time of STEAM the diffusion becomes more anisotropic as ECV decreases for all geometry types, varying approximately linearly at different rates depending on the geometry. With the spin echo sequences, the sensitivity of FA to ECV is relatively minor. During thickening, FA varies by 0.027 for PGSE and 0.054 for M2‐SE. Shrinking the cardiomyocytes substantially reduces FA for PGSE and STEAM, but M2‐SE is almost unaffected (change of only 0.020).

**Figure 7:**
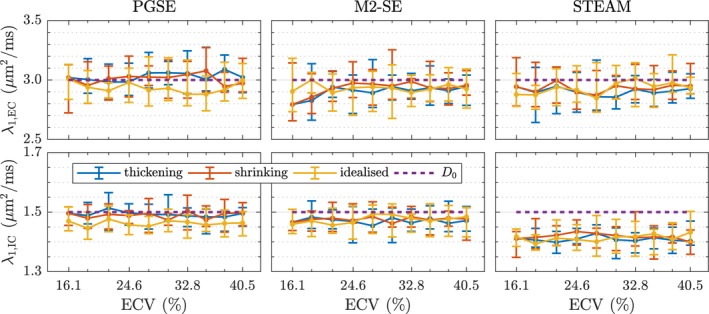
Separating the signal from particles in the two separate intra‐ and extra‐cellular compartments shows the sensitivity of the three pulse sequences to unrestricted diffusion along the first eigenvector. Each data point is the median value obtained from 10 realisations and the error bars indicate the two‐sided 95% confidence interval

Since the simulations were performed with impermeable boundaries, it is possible to separate the effect of intra‐ and extra‐cellular changes in the microstructure. In Figure 7 we plot λ_1_ for tensors obtained separately from particles in the two compartments. There is no dependence on ECV or shape of the cells in the given simulation substrate. The prescribed diffusivities of *D*_EC_ = 3.0 μm^2^/ms and *D*_IC_ = 1.5 μm^2^/ms are recovered almost exactly by PGSE and M2‐SE, but with STEAM there is an offset of approximately −0.1 and −0.2 μm^2^/ms for extra‐ and intra‐cellular λ_1_ respectively.

### 3.4 Cone of uncertainty analysis

**Figure 8:**
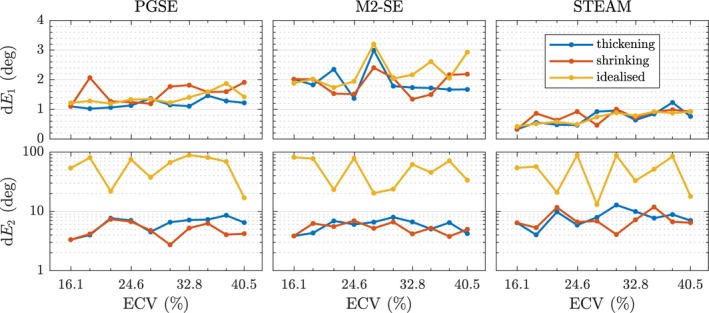
The 95% one‐sided confidence interval of the error in first and second eigenvector orientation shows the uncertainty of each pulse sequence

The 95% confidence intervals on the primary and secondary eigenvector orientations (d*E*_1_, d*E*_2_) are plotted in Figure 8 for the three sequences and geometry types. The uncertainty of d*E*_1_ is largest for M2‐SE with an average of 2.0^∘^. The PGSE sequence exhibits a maximum uncertainty of 2.0^∘^ for high ECV values and 1.0^∘^ for ECV of 16%. Uncertainty of the STEAM sequence is the lowest but increases with ECV.

The cone of uncertanty around the secondary eigenvector (d*E*_2_) is insensitive to ECV or pulse sequence, ranging from 2.0^∘^ to 11.0^∘^ for the histology‐based substrates. The idealised geometry shows a large uncertainty of up to 90^∘^ for d*E*_2_.

### 3.5 Effect of maximum gradient strength

**Figure 9:**
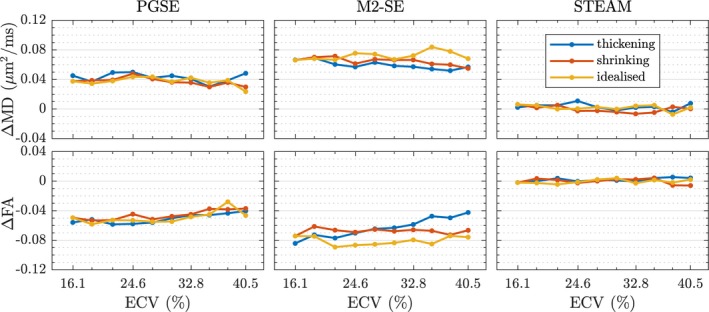
The effect of using a stronger maximum gradient strength (*G*_max_) is visualised by comparing mean diffusivity and fractional anisotropy results. Each data point is the difference between median values obtained for the two gradient strengths 40 and 80 mT/m, i.e. Δ*y* = *y*_80_−*y*_40_

To investigate how a different gradient strength may affect the sensitivity of sequences to the microstructural parameters, we compare the results for *G*_max_ = 40 mT/m with those obtained for *G*_max_ = 80 mT/m. In Figure 9 the differences between MD and FA are shown. Positive values of ΔMD indicate higher observed MD with 80 mT/m than with 40 mT/m using both PGSE and M2‐SE. Similarly, negative values of ΔFA indicate lower FA at the higher gradient strength for the spin echo sequences. STEAM sequence shows no noticeable sensitivity to gradient strength.

## 4 Discussion

### 4.1 Key findings

We have demonstrated the feasibility of performing Monte Carlo random walk simulations of DT‐CMR with a histology‐based microstructure and realistic imaging voxel size for typical in‐vivo pulse sequences. These included both short and long diffusion times (M2‐SE and STEAM) and we compared the results to those from a PGSE sequence for reference. This extends recent work simulating ex‐vivo DT‐CMR,^29^ where the authors simulated a Stejskal–Tanner PGSE sequence in a cuboid‐based simulation substrate with 200 μm isotropic voxel size.

To carry out the experiments in this work we have developed an efficient parallel random walk algorithm capable of handling arbitrary cell geometries. Convergence tests in in Supporting Information Figure S1 show that 10 repetitions per data point using *N*_P_ = 10^4^ and *N*_T_ = 10^3^ is sufficient to reliably compute DT‐CMR parameters while keeping the simulation within an acceptable runtime. These findings are consistent with the observations in simulations of cardiac^29^ and brain tissue.^48^ The normally‐distributed step lengths aid convergence and a rejection threshold of ±5σ is sufficient for accuracy (Supporting Information Figure S2). Since the Monte Carlo random walk is efficiently parallelised the factors that determine simulation runtime are the number of particles, the per‐particle computational cost, and the processor speed and number of available threads. We have found the per‐particle computational cost to scale with both *N*_T_ and the number of faces (*N*_F_) used to represent the cell geometry, see Supporting Information Figure S3. Idealised myocytes, modelled as cuboids, have few faces and therefore can be simulated in a fraction of the time needed for the histology‐based geometry. However, we have also shown that, despite matching the average dimensions and ECV, these simplified geometries do not provide equivalent results to the histology‐based simulations.

In the past, other groups have used various methods to synthesise DTI data. The diffusion MRI software package Camino^49^ has been used to simulate a wide range of geometries with applications to the brain.^25^, ^48^, ^50^ However, these studies lack the long diffusion times and microstructural architecture characteristic of DT‐CMR. Another approach is to use available packages to simulate the motion of molecules (Smoldyn^51^ as done by,^29^ or MCell^52^ as done by^30^) and synthesise the MR signal by applying the pulse sequence during post‐processing. This may come at a large overhead in computation or software development. Others have used problem‐specific software to study parameters of interest, for example^28^ in which the authors investigate the dependence of apparent diffusion coefficient (ADC) on neuronal cell size and perform simulations of fibre bundle configurations such as crossing or branching. 27used bundles of tightly‐packed hexagonal cylinders to represent cardiomyocytes, which ignored the inhomogeneity and structure of ECS and lacked the large domain size of typical DT‐CMR voxels.

By creating the simulation substrate directly from histology we are able to retain the distribution of extra‐cellular space, and importantly the shear layers, in our model. Recently, 30used histology data of skeletal muscle tissue to show that simple hexagonal arrays of cells may be used to simulate realistic microstructure. However, in this work, we show that the complex structure of the heart is not adequately represented by a simple cuboidal representation. Through our proposed method, we are able to augment the cells in the histology‐based geometry directly and thereby both compensate for tissue shrinkage during fixation as well as investigate the effect of ECV and ECS distribution on DT‐CMR parameters.

We progressively morphed (by thickening and then shrinking) the cardiomyocytes. As stated in recent in‐vivo studies^12^, ^53^ the apparent diffusion coefficient is correlated with ECV. We observe this too, as seen in the both λ_1_ and MD plots in Figure 6 with high *R* ^2^ values for all three pulse sequences. This is logical, since a lower ECV means more particles are located in intra‐cellular space where the diffusivity is lower; since the signal contribution is weighted equally, ADC will be lower regardless of the effect of barriers. The observed eigenvalues and MD may be compared to the volume‐weighted free diffusivity

(5) $$〈D_{0}〉\;=\;\text{ECV}\times D_{\text{EC}}+\left(1-\text{ECV}\right)\times D_{\text{IC}}.$$

This is the theoretical ADC value if no barriers are present, and thus corresponds to the case of high permeability and/or very short mixing times, or low *b*‐value. It is therefore indicative of how well the different sequences probe the microstructure and sense restriction. The first eigenvalue λ_1_ in Figure 6 shows a slightly lower value than $\left\langle D_{0}\right\rangle$. Since the corresponding eigenvector ${\vec{E}}_{1}$ is aligned with the direction of least restriction, the difference is likely attributed to particles near the cardiomyocyte intercalated discs. This is primarily due to restriction on IC particles, as *D*_IC_ is reduced in Figure 7. The reduced MD values are the result of significantly lower values of λ_2_ and λ_3_ (compare Supporting Information Figure S7), even for spin‐echo sequences—a result of the relatively small cross‐section of myocytes. For STEAM this difference is larger, showing how a longer mixing time “feels” the restriction more and reduces ADC.

There is no clear common trend among sequences in how FA changes with variations in ECV. In the case of STEAM, particles diffuse for a relatively long time (Δ = 1 s) and potentially cover a large volume. Those particles located inside cardiomyocytes can thus cover the entire cell shape and sense all its boundaries. The pencil‐shaped nature of the geometry causes a large FA. Spin‐echo sequences on the other hand have a short diffusion time. While it is difficult to define Δ precisely for the M2‐SE sequence due to the asymmetric gradient shapes, generously assuming a diffusion time of 36 ms from the start of the first gradient to the start of the first diffusion gradient after the 180^∘^ pulse, gives a mean displacement of $\sqrt{6D_{\text{IC}}\Delta }\;=\;18\;\mu$m for M2‐SE and *D*_IC_ = 1.5 μm^2^/ms. Given that the typical cardiomyocyte diameter is around the same as this displacement or slightly larger, the restriction that IC particles experience is low, reducing measured diffusion anisotropy. When the myocytes are thickened there are even fewer IC restrictions, further reducing FA. For EC particles, the restriction increases as the gaps between cells shrink. Our results suggest that these two effects somewhat cancel each other out, leading to only modest changes in FA for both spin‐echo sequences as ECV is varied. Comparing the results for M2‐SE with those for PGSE, we notice that the former seems to have a lower effective diffusion time based on a higher MD and lower FA obtained with this sequence.

Figure 6 shows, interestingly, that MD and FA vary between geometries with the same ECV but a different distribution of ECS as a result of changes in shape of the cardiomyocytes due to morphing. Initially, the intra‐sheetlet ECS is non‐uniform and large spaces as well as thin gaps are present. When thickening the myocytes, pockets of ECS are created between them as the smallest gaps quickly close, making it difficult for particles to escape. This is in contrast to the behaviour when the segmentation map with ECV = ECV_min_ is being morphed to increase ECV. When shrinking the myocytes, the gaps in the ECS that were just one pixel wide grow uniformly everywhere. As a result, it becomes easier for extra‐cellular particles to move around thereby increasing MD and lowering FA. This is observed regardless of pulse sequence and suggests that the non‐uniform cardiomyocyte spacing maintained with our thickening algorithm is important for accurate simulations.

The large size of a typical DT‐CMR voxel (millimetre scale), necessary to achieve a sufficient SNR within an acceptable duration, means that the tissue (micrometre scale) is highly inhomogeneous throughout the voxel. For example, in our simulation substrate the myocyte orientation varied by 24^∘^ in the transmural direction. While the mean orientation (primary eigenvector directions) will be recovered correctly when using a large voxel size, the fanning of the cardiomyocytes within the voxel^22^, ^54^ affects the diffusion tensor, especially FA. Results in Figure 6 showed that λ_1_ does not exhibit the observed dependence on ECS distribution and its variation with ECV is nearly linear.

We calculated the cone of uncertainty in Figure 8. The results show that the long mixing time of STEAM helps in reducing the uncertainty on the ${\vec{E}}_{1}$. In comparison, the shorter spin echo sequences exhibit larger uncertainty, with M2‐SE having the largest d*E*_1_. This suggests that, while difficult to define, M2‐SE has a lower effective diffusion time. This is corroborated by the reduced FA compared to that of PGSE. The dependence on ECV shows that the uncertainty is due to the fact that ECS is a large hindered (but not restricted) compartment, whereas ICS has a clear orientation associated with each cardiomyocyte. There was no significant effect when using a higher gradient strength (compare Supporting Information Figures S8 and S9).

When considering d*E*_2_, there is no clear difference between sequences. However, we find a large uncertainty on ${\vec{E}}_{2}$ for the idealised geometry. Random or regular arrays of cuboids as used in this work have no structural disorder in ECS. The sheetlet structures found in the myocardium therefore reduce uncertainty on the secondary eigenvector of the diffusion tensor.

The purpose of this initial work was to establish the methodology necessary to carry out further investigations, enabling the simulation of more realistic geometries based on histology. While the changes in ECV are not pathological and instead attempt to compensate for and determine the sensitivity of results to tissue shrinkage, the findings presented here suggest potentially interesting clinical applications. By comparing results in Figures 5 and 6 we find that the sensitivity of MD to changes in diffusivity is much larger than to ECV. From this we might hypothesise that any significant change in observed MD, beyond what can be accounted for by in‐vivo estimates of ECV, is likely due to (pathological) changes in *D*_IC_. We also showed that the effect of changes in *D*_EC_ may be observed in MD but not FA.

### 4.2 Limitations

The simulation substrate used in this study was constructed based on a single histological block from one pig. A sub‐voxel‐sized region in one slice of wide‐field microscopy images was manually segmented. The simulation voxel thus consists of copies of one 500 × 400 × 120 μm^3^ block with ca. One thousand myocytes each. By stacking these with an offset and applying the sinusoidal displacement, we have reduced the effect of extra‐cellular gaps running along the entire voxel. The prescribed HA creates tissue inhomogeneity and ensures there are 7 additional rotated versions of this building block. Due to constraints with stacking blocks, cardiomyocytes are assumed to be parallel within each building block and hence any three‐dimensional nature of the sheetlets may be lost. Recent work has found cardiomyocyte curvature to be 2^∘^/mm.^55^ Since DTI is rotationally invariant, resultant changes around ${\vec{E}}_{1}$ should be small. The effect of intra‐voxel fibre dispersion and myocyte branching is assumed to be minimal, but if implemented may lead to a decrease in anisotropy. To generate more realistic 3D geometries, sheetlets or cardiomyocytes would need to be semi‐automatically segmented in‐plane and traced along image stacks, which is a substantial additional task.

While the histology used in this study was of a very high quality it suffers from tissue shrinkage as a result of fixation and possible distortion during sectioning. By thresholding the histology ROI segmented in this work, an extra‐cellular area fraction of 38% can be estimated. This is higher than expected for the myocardium^53^, ^56^ and therefore techniques such as the cardiomyocyte thickening and shrinking algorithms described in this work must be used. We have demonstrated that the precise method in which this is done affects the effective restriction of particle diffusion and that the non‐uniform spacing of cardiomyocytes should be maintained in virtual microstructures. Additionally, the sectioning methods may cause sheetlets to lose cohesion and result in an increase in the size of shear layers. This was not addressed in this research, since the cells were prevented from entering the cleavage planes during morphing. Future work may attempt to compensate for this effect when generating the virtual microstructure, but needs to ensure sufficient space between sheetlets. Other, less destructive imaging methods, including synchrotron X‐ray phase contrast CT,^57^ could also be incorporated to obtain histology data more representative of in‐vivo tissue.

In the model used here, we have assumed a isotropic homogeneous diffusivity within each of the two compartments. However, the ECS contains both collagen and microvasculature and cardiomyocytes have a nanoscale internal structure which may restricts diffusion. By using a reduced diffusivity of *D*_IC_ = 1.5 μm^2^/ms within the cardiomyocytes, we model the cytosol as a homogeneous medium and therefore neglect the nanoscale structure of the cells. There exists very little literature on the free diffusivity coefficient in the cardiac tissue. 58identified two compartments in rat hearts through ex‐vivo experiments at 37^∘^C. The observed values were 1.2 and 3.0μm^2^/ms, presumably intra‐ and extra‐cellular diffusivities respectively. 29used constant *D*_IC_ = *D*_EC_ when modelling cardiac tissue, and^26^ varied the ratio of *D* and compared results to the Kärger model^59^ for simulations of white matter.

Recent work^60^, ^61^ suggests that the high vascular volume of the myocardium may impact overall MD and FA. In our model we have neglected perfusion and modelled the ECS as a homogeneous medium. The inclusion of perfusion would likely increase MD. Particles being advected by the flow inside microvessels add a fast signal in the voxel. Due to the alignment of vasculature with the surrounding cells,^62^, ^63^ this may increase FA as demonstrated by.^64^ However, generally a non‐zero reference b‐value is used in vivo, which minimises the effect of perfusion^61^, ^65^ and simulations would need to use the same pulse sequence and b‐value combinations. Due to a lack of data on flow speed and large‐scale vessel architecture, it is currently not possible to construct a fully‐realistic substrate. Wide‐field microscopy images of pig heart lack most extra‐cellular tissue features, including microvasculature, but future work may use confocal microscopy such as that described in^62^, ^63^ to inform more complex models. This is essential to further reduce the gap between in‐silico and in‐vivo tissue.

Unlike in‐vivo experiments, the geometry model in the simulations was static. During the cardiac cycle, cardiomyocytes contract and relax, thereby changing shape. It is unclear if the intra‐cellular diffusivity changes as a result of cell strain or other confounding factors.

The random walk model we implemented assumes impermeable boundaries. As others^25^, ^26^, ^29^, ^66^ have found, the effect of permeability is limited and may be neglected. However, past work has not included the long mixing times inherent in the STEAM sequence. The mean displacement in the extra‐cellular space in such a case is $\sqrt{6D_{e}T}\;=\;134\;\mu$m, which covers multiple cell diameters. Permeable membranes may therefore reduce FA for the case of STEAM, to levels typically observed in vivo for this sequence.^41^, ^65^ In the study by,^27^ the authors also report a high FA of up to 0.8 in‐silico, despite the short diffusion time of 64 ms.

Other factors that may contribute to the differences in DT‐CMR parameters between these simulations and in‐vivo imaging include possible active transport across cell membranes and facilitated transport of water due to other processes such as the contraction of the myofilaments. Differences between intra‐ and extra‐cellular relaxation parameters may also influence in‐vivo DT‐CMR results and could be included in future simulations. This is a topic of future investigation, which first requires the methodology developed in the current work to reduce model uncertainties surrounding the microstructural geometry.

## 5 Conclusions

A realistic DT‐CMR voxel has a number of interspersed sheetlet and myocyte populations, and cardiomyocytes have an irregular morphology with complicated shapes (e.g. due to branching), and variable sizes. However, literature often models them as simple shapes such as cylinders or cuboids in dense arrangement. This work has shown that the distribution of ECS has a measurable impact on DT‐CMR parameters. We have also demonstrated a method for morphing a given segmentation map to compensate for e.g. tissue shrinkage. This may be used in the future to develop simple models consisting of analytical shapes, whilst respecting the global microstructural arrangement.

The computational cost of arbitrarily‐shaped myocytes, which stems from their high number of surface mesh faces, is a big drawback which limits the parameter space covered during sensitivity studies. We have shown that our efficient random walk algorithm performs well on idealised shapes. A next direction is the development of models consisting of simple shapes in a histology‐based arrangement, to combine the computational efficiency of a low number of mesh faces with a realistic model of ECS. Future applications include the rapid simulation of DT‐CMR pulse sequences with varying microstructural model and sequence parameters. This would generate large datasets and meaningful statistics. In this work we have studied the uncertainty and found a dependence on the underlying geometry, albeit non‐pathological. The future capability may be exploited to investigate the sensitivity and minimise uncertainty of current and future DT‐CMR pulse sequences to disease by studying the effect of microstructural changes and the benefits of e.g. b‐values or gradient strengths.

## Supporting information


**Figure S1** Convergence of mean diffusivity (MD) and fractional anisotropy (FA) with increasing number of particles (*N*_P_) and number of timesteps (*N*_T_) for all three pulse sequences
**Figure S2** Convergence of mean diffusivity (MD) and fractional anisotropy (FA) with increasingly larger rejection threshold on the normally‐distributed random step length for all three pulse sequences. Both free diffusion and a histology‐based substrate are simulated. For the former, we do not plot difference in FA due to the large relative error near FA = 0
**Figure S3** Increase in computational cost calculated as the ratio of runtime (RT) to the initial runtime (RT_0_). Left: Two “dummy” pulse sequences with *G*(*t*) = 0 and constant step size d*t* = *T*/*N*_T_ over duration *T* with a varying number of timesteps (*N*_T_) were simulated. Right: The faces of all cuboids in the idealised geometry were subdivided to increase the total number of faces (*N*_F_)
**Figure S4** Schematic diagrams of the three pulse sequences, normalised in time by the total time *T*. Annotations show the definition of the characteristic sequence parameters where appropriate. All gradients are symmetrical
**Figure S5** Diffusion tensor parameters as a function of intra‐cellular diffusivity (*D*_IC_) for two values of extra‐cellular diffusivity (*D*_EC_). The substrate was a histology‐based geometry with ECV = 25% and *G*_max_ = 40 mT/m. The units for λ_1_, λ_2_, λ_3_, and MD are μm^2^/ms and those for d*E*_1_, d*E*_2_, and d*E*_3_ are deg
**Figure S6** Diffusion tensor parameters as a function of intra‐cellular diffusivity (*D*_IC_) for two values of extra‐cellular diffusivity (*D*_EC_). The substrate was a histology‐based geometry with ECV = 25% and *G*_max_ = 80 mT/m. The units for λ_1_, λ_2_, λ_3_, and MD are μm^2^/ms and those for d*E*_1_, d*E*_2_, and d*E*_3_ are deg
**Figure S7** Diffusion tensor parameters as a function of extra‐cellular volume fraction (ECV) and geometry type for *G*_max_ = 40 mT/m and diffusivity values of 1.5 and 3.0 μm^2^/ms, intra‐cellular diffusivity (*D*_IC_) and extra‐cellular diffusivity (*D*_EC_) respectively. The units for λ_1_, λ_2_, λ_3_, and MD are μm^2^/ms and those for d*E*_1_, d*E*_2_, and d*E*_3_ are deg
**Figure S8** Diffusion tensor parameters as a function of extra‐cellular volume fraction (ECV) and geometry type for *G*_max_ = 40 mT/m and diffusivity values of 1.0 and 2.0μm^2^/ms, intra‐cellular diffusivity (*D*_IC_) and extra‐cellular diffusivity (*D*_EC_) respectively. The units for λ_1_, λ_2_, λ_3_, and MD are μm^2^/ms and those for d*E*_1_, d*E*_2_, and d*E*_3_ are deg
**Figure S9** Diffusion tensor parameters as a function of extra‐cellular volume fraction (ECV) and geometry type for *G*_max_ = 80 mT/m and diffusivity values of 1.0 and 2.0 μm^2^/ms, intra‐cellular diffusivity (*D*_IC_) and extra‐cellular diffusivity (*D*_EC_) respectively. The units for λ_1_, λ_2_, λ_3_, and MD are μm^2^/ms and those for d*E*_1_, d*E*_2_, and d*E*_3_ are deg
