# Supplementary material for: Novel insights into in‐vivo diffusion tensor cardiovascular magnetic resonance using computational modelling and a histology‐based virtual microstructure
Source: Magn Reson Med. 2018 Oct 23;81(4):2759–73. doi: 10.1002/mrm.27561 (PMC6637383; doi:10.1002/mrm.27561)
Supplement: Supplementary file 1 — Figure S1 Convergence of mean diffusivity (MD) and fractional anisotropy (FA) with increasing number of particles (N P) and number of timesteps (N T) for all three pulse sequences Figure S2 Convergence of mean diffusivity (MD) and fractional anisotropy (FA) with increasingly larger rejection threshold on the normally‐distributed random step length for all three pulse sequences. Both free diffusion and a histology‐based substrate are simulated. For the former, we do not plot difference in FA due to the large relative error near FA = 0 Figure S3 Increase in computational cost calculated as the ratio of runtime (RT) to the initial runtime (RT0). Left: Two “dummy” pulse sequences with G(t) = 0 and constant step size dt = T/N T over duration T with a varying number of timesteps (N T) were simulated. Right: The faces of all cuboids in the idealised geometry were subdivided to increase the total number of faces (N F) Figure S4 Schematic diagrams of the three pulse sequences, normalised in time by the total time T. Annotations show the definition of the characteristic sequence parameters where appropriate. All gradients are symmetrical Figure S5 Diffusion tensor parameters as a function of intra‐cellular diffusivity (D IC) for two values of extra‐cellular diffusivity (D EC). The substrate was a histology‐based geometry with ECV = 25% and G max = 40 mT/m. The units for λ1, λ2, λ3, and MD are μm2/ms and those for dE 1, dE 2, and dE 3 are deg Figure S6 Diffusion tensor parameters as a function of intra‐cellular diffusivity (D IC) for two values of extra‐cellular diffusivity (D EC). The substrate was a histology‐based geometry with ECV = 25% and G max = 80 mT/m. The units for λ1, λ2, λ3, and MD are μm2/ms and those for dE 1, dE 2, and dE 3 are deg Figure S7 Diffusion tensor parameters as a function of extra‐cellular volume fraction (ECV) and geometry type for G max = 40 mT/m and diffusivity values of 1.5 and 3.0 μm2/ms, intra‐cellular diffusivity (D IC) and extra‐cellular diffu [file MRM-81-2759-s001.pdf]

Statistical Convergence

Monte Carlo parameter choice

A sufficient number of particles (N_P) and number of timesteps (N_T) is crucial to achieve converged repeatable results. Since the simulation runtime scales linearly with the number of particles, a trade-off is necessary to keep the computational cost at an acceptable level and increase the amount of model parameters that can be investigated. A convergence study was carried out to ensure a sufficient number of particles are simulated. We assume a sufficiently converged solution at $N_\mathrm{P} = 10^5$ and $N_\mathrm{T} = 10^4$ and evaluate the error of using less particles and timesteps. Simulations were performed for a histology-based substrate with ECV = 25 %, $D_\mathrm{IC} = 1.5\,\mu\mathrm{m}^2/\mathrm{ms}$, and $D_\mathrm{EC} = 3.0\,\mu\mathrm{m}^2/\mathrm{ms}$.

The variance of extracted tensor parameters for repeated experiments decreases with an increase in the number of particles. When plotting the data points for all 10 realisations as a function of N_P, done in Supporting Information Figure S1, we observe that the maximum absolute deviation of mean diffusivity (MD) and fractional anisotropy (FA) from the mean is generally less than 2.5 % for $N_\mathrm{P} = 10^4$ and $N_\mathrm{T} = 10^3$. Similar values were observed for the individual eigenvalues (not shown here). A finer timestep, i.e. $N_\mathrm{T} = 10^4$, only slightly reduced this range.

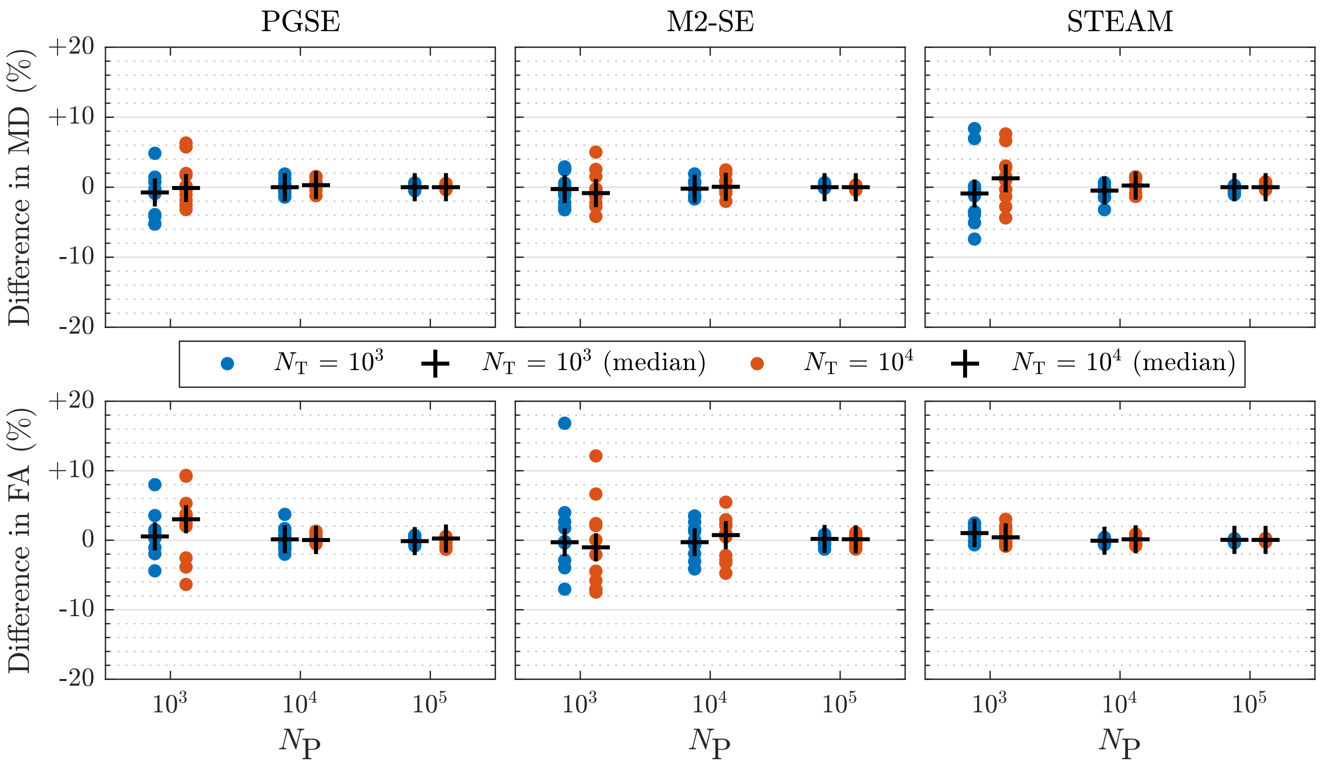

Supporting Information Figure S1: Convergence of mean diffusivity (MD) and fractional anisotropy (FA) with increasing number of particles (N_P) and number of timesteps (N_T) for all three pulse sequences.

Random walk step size limit

A larger step length increases the search space of possible boundary intersections. Because each particle's displacement vector R has a normally-distributed random length, it is possible that it will require an excessive amount of sub-steps to resolve the step. While a Monte Carlo process requires this to be included, there is only a small error associated with discarding rare large steps for the benefit of reduced computational complexity. In Supporting Information Figure S2 we show the effect of setting a different step rejection threshold.

We also compare the results from the histology-based substrate to that of free diffusion. The latter is a good test case to verify the parameter choice as each pulse sequence should recover free diffusion with little error. As can be seen, the median error for 5σ is less than 1 % for all parameters and sequences.

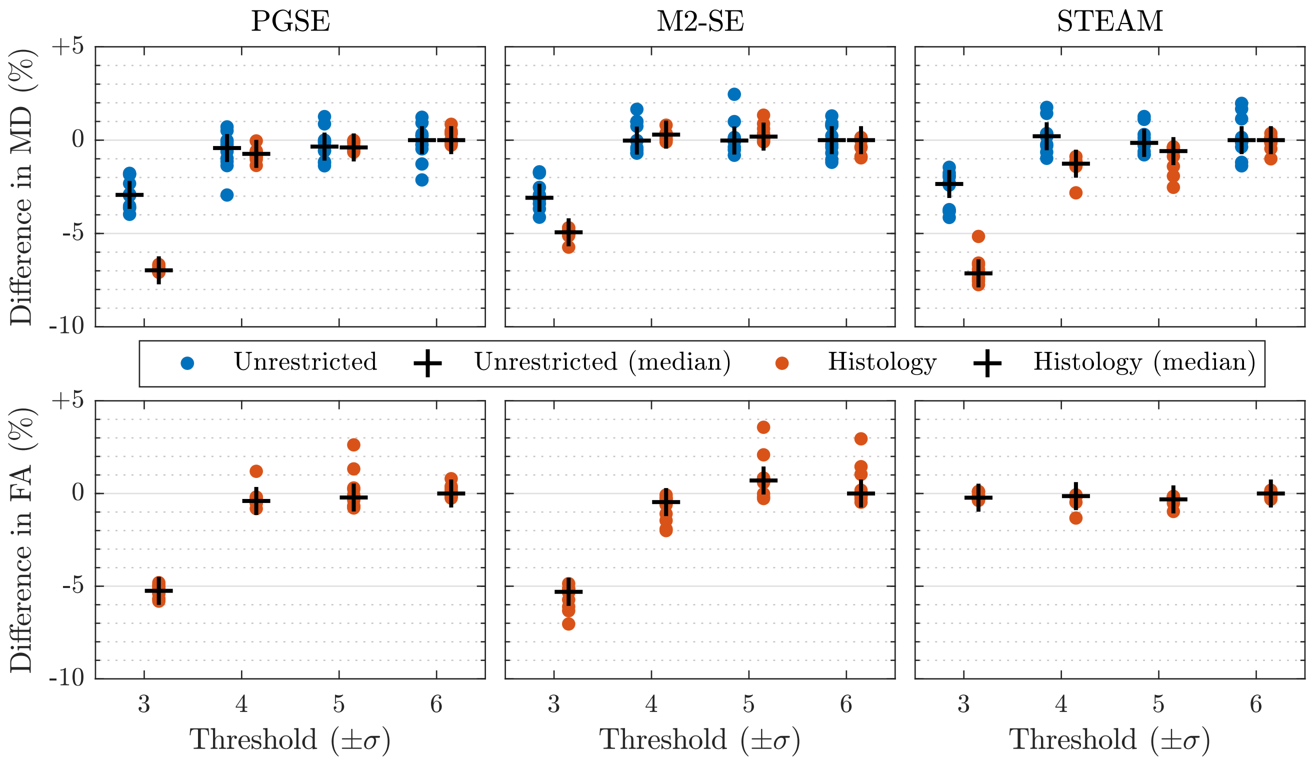

Supporting Information Figure S2: Convergence of mean diffusivity (MD) and fractional anisotropy (FA) with increasingly larger rejection threshold on the normally-distributed random step length for all three pulse sequences. Both free diffusion and a histology-based substrate are simulated. For the former, we do not plot difference in FA due to the large relative error near FA $= 0$.

Runtime

Using $N_\mathrm{P} = 10^4$ and $N_\mathrm{T} = 10^3$ resulted in an average runtime of 1.8 h per sample for spin echo sequences and 6.8 h for STEAM. Despite using the same number of timesteps and particles in all sequences, the long mixing time and corresponding larger proportion of long timesteps requires more sub-steps for STEAM. Since the highest computational cost lies with computing intersections, the runtime almost directly scales with number of sub-steps rather than timesteps. The simulation runtime was orders of magnitude shorter for the simple cuboid geometry, averaging about 3 min. Because each triangular mesh representing a cuboid has only 8 vertices and 12 triangular faces, even a naïve mesh-based treatment is significantly less computationally expensive than arbitrarily-shaped myocytes.

We observe for $T = 1.0\,\mathrm{s}$ a runtime slightly larger than for $T = 0.1\,\mathrm{s}$, due to the longer step size $\mathrm{d}t$ and corresponding increase in sub-steps. When simulating a substrate with more mesh faces, the runtime increases non-linearly but monotonically.

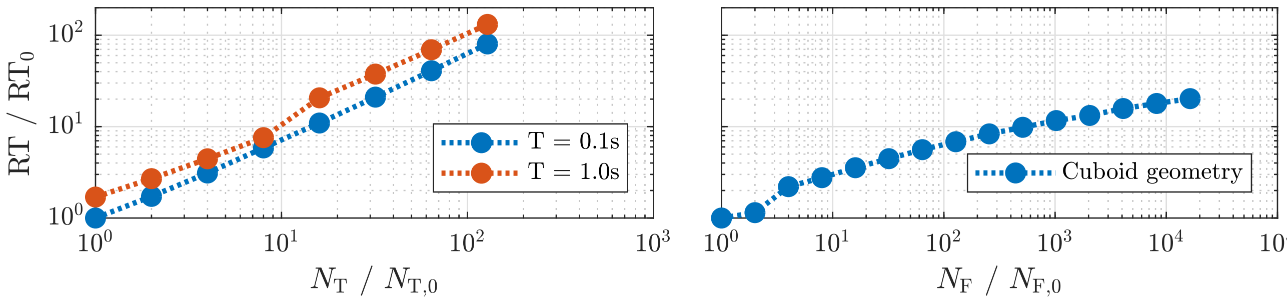

Supporting Information Figure S3: Increase in computational cost calculated as the ratio of runtime (RT) to the initial runtime (RT_0). Left: Two "dummy" pulse sequences with $G(t) = 0$ and constant step size $\mathrm{d}t = T/N_\mathrm{T}$ over duration T with a varying number of timesteps (N_T) were simulated. Right: The faces of all cuboids in the idealised geometry were subdivided to increase the total number of faces (N_F).

Pulse sequence diagrams

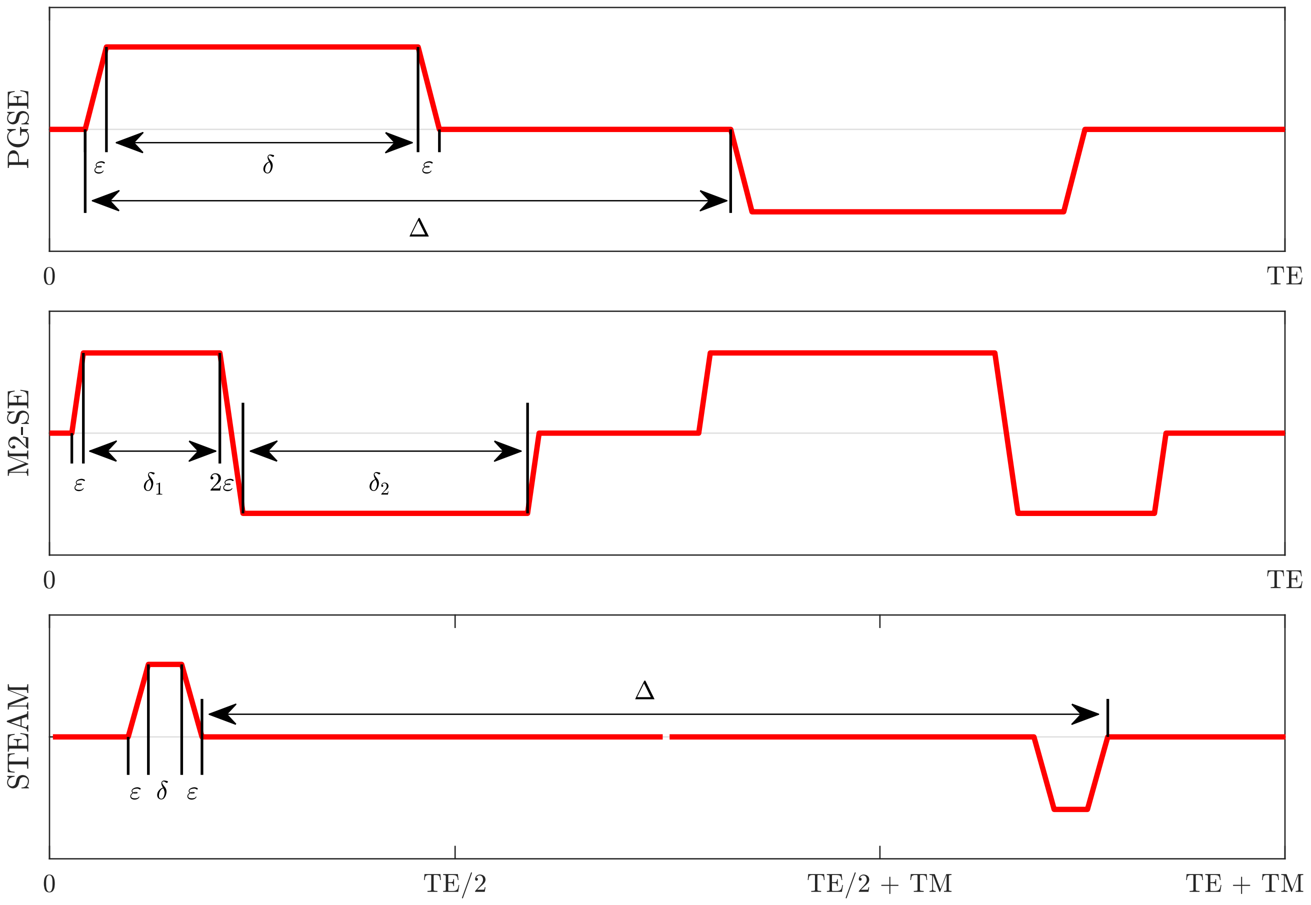

Supporting Information Figure S4: Schematic diagrams of the three pulse sequences, normalised in time by the total time T. Annotations show the definition of the characteristic sequence parameters where appropriate. All gradients are symmetrical.

Varying compartment-specific diffusivities

See Supporting Information Figures S5 and S6.

Varying the extra-cellular volume fraction

See Supporting Information Figures S7 to S9.

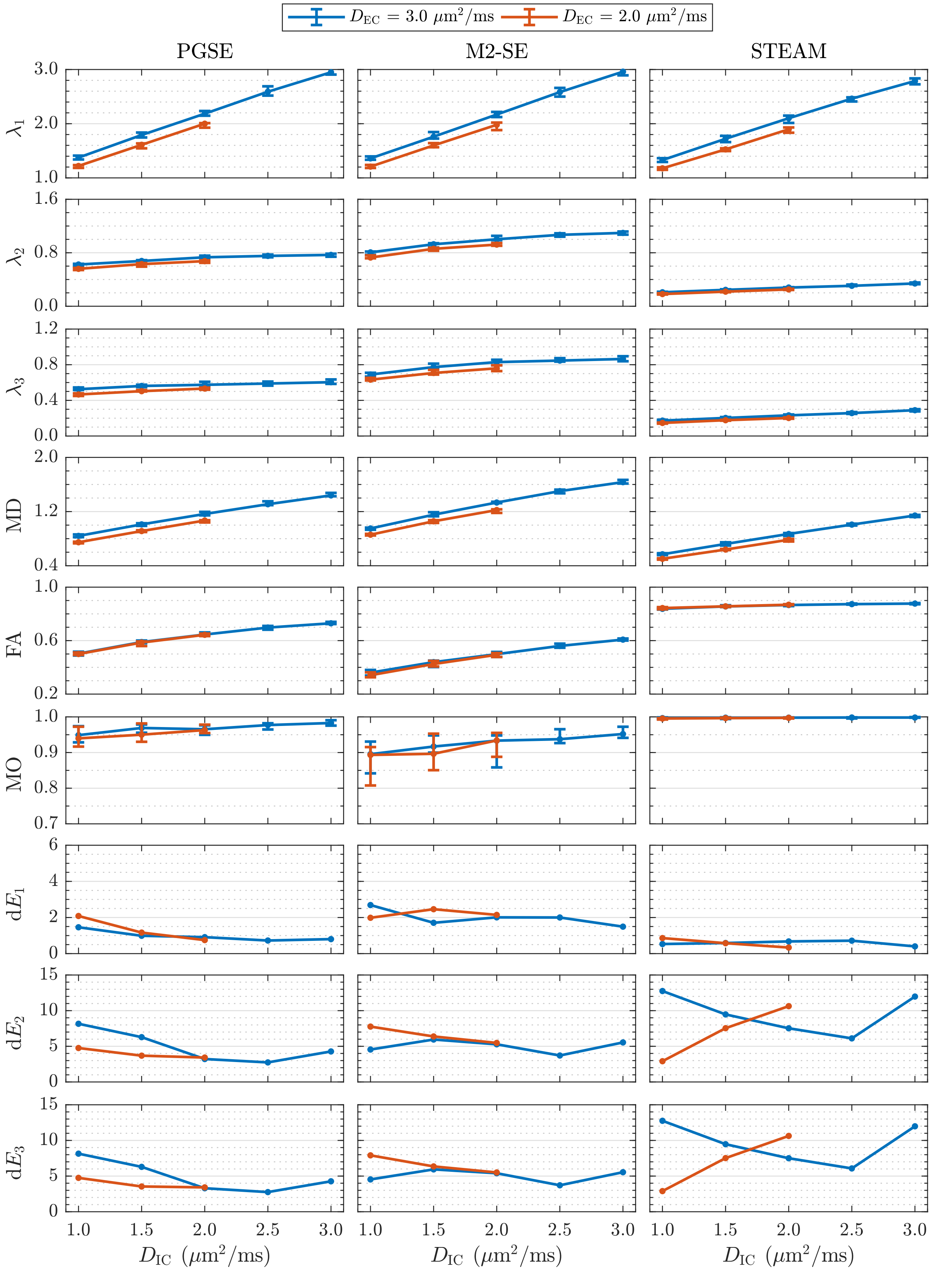

Supporting Information Figure S5: Diffusion tensor parameters as a function of intra-cellular diffusivity (D_{IC}) for two values of extra-cellular diffusivity (D_{EC}). The substrate was a histology-based geometry with ECV = 25 % and G_{max} = 40 mT/m. The units for λ_1, λ_2, λ_3, and MD are μm²/ms and those for dE_1, dE_2, and dE_3 are deg.

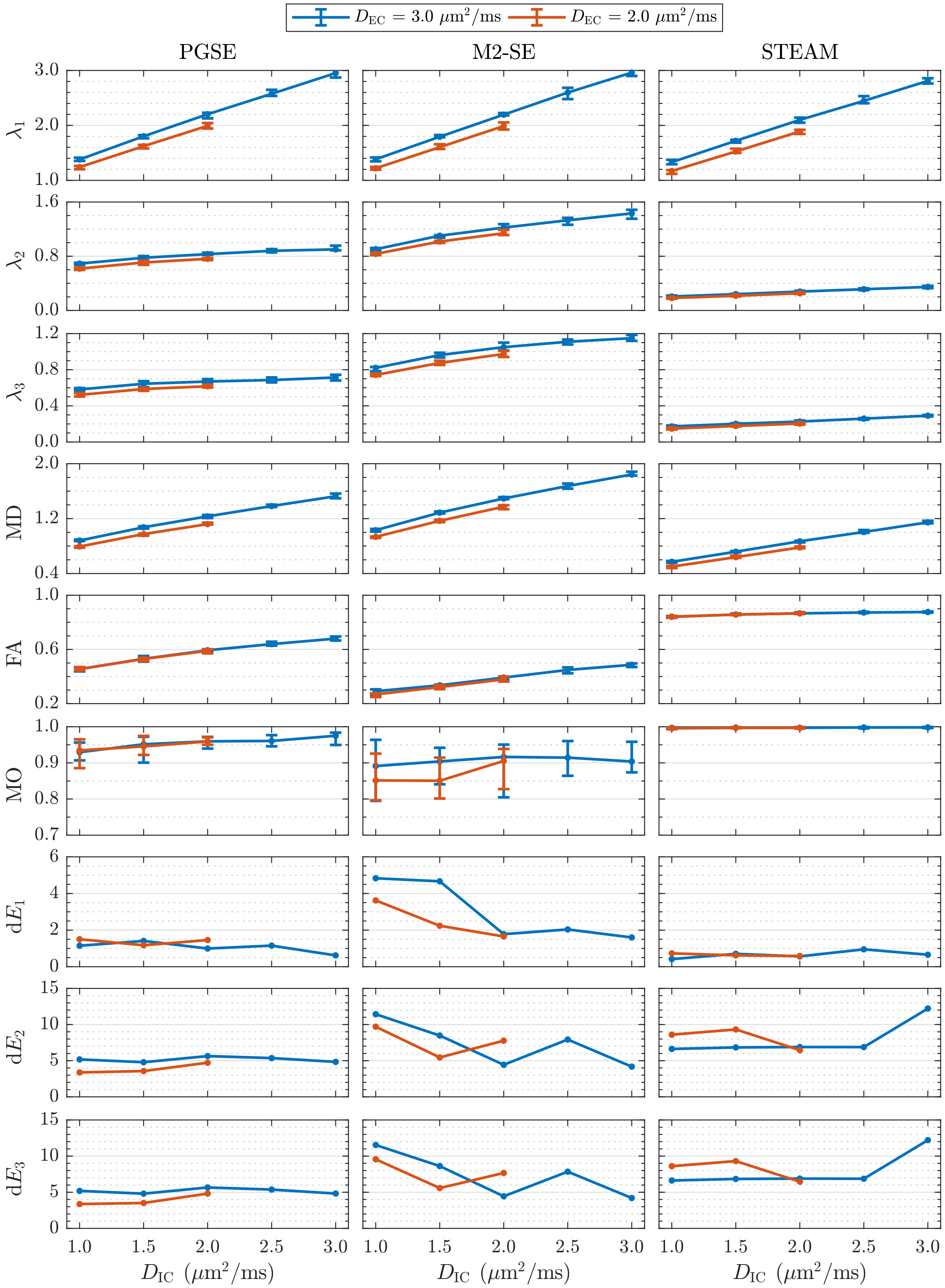

Supporting Information Figure S6: Diffusion tensor parameters as a function of intra-cellular diffusivity (D_{IC}) for two values of extra-cellular diffusivity (D_{EC}). The substrate was a histology-based geometry with ECV = 25 % and G_{max} = 80 mT/m. The units for λ_1, λ_2, λ_3, and MD are µm^2/ms and those for dE_1, dE_2, and dE_3 are deg.

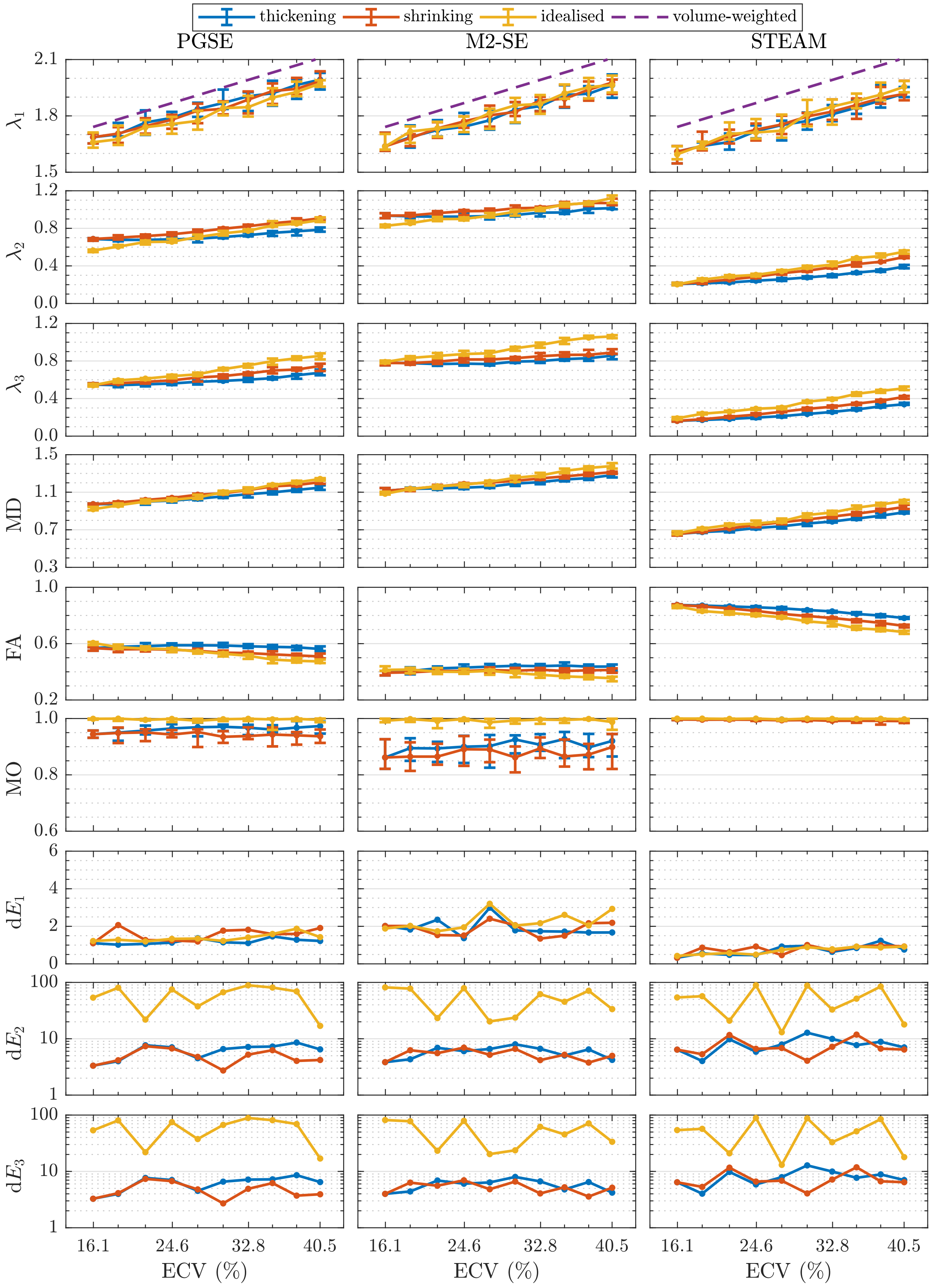

Supporting Information Figure S7: Diffusion tensor parameters as a function of extra-cellular volume fraction (ECV) and geometry type for $G_{\max} = 40\,\mathrm{mT/m}$ and diffusivity values of 1.5 and $3.0\,\mu\mathrm{m}^2/\mathrm{ms}$, intra-cellular diffusivity (D_{IC}) and extra-cellular diffusivity (D_{EC}) respectively. The units for λ_1, λ_2, λ_3, and MD are $\mu\mathrm{m}^2/\mathrm{ms}$ and those for $\mathrm{d}E_1$, $\mathrm{d}E_2$, and $\mathrm{d}E_3$ are deg.

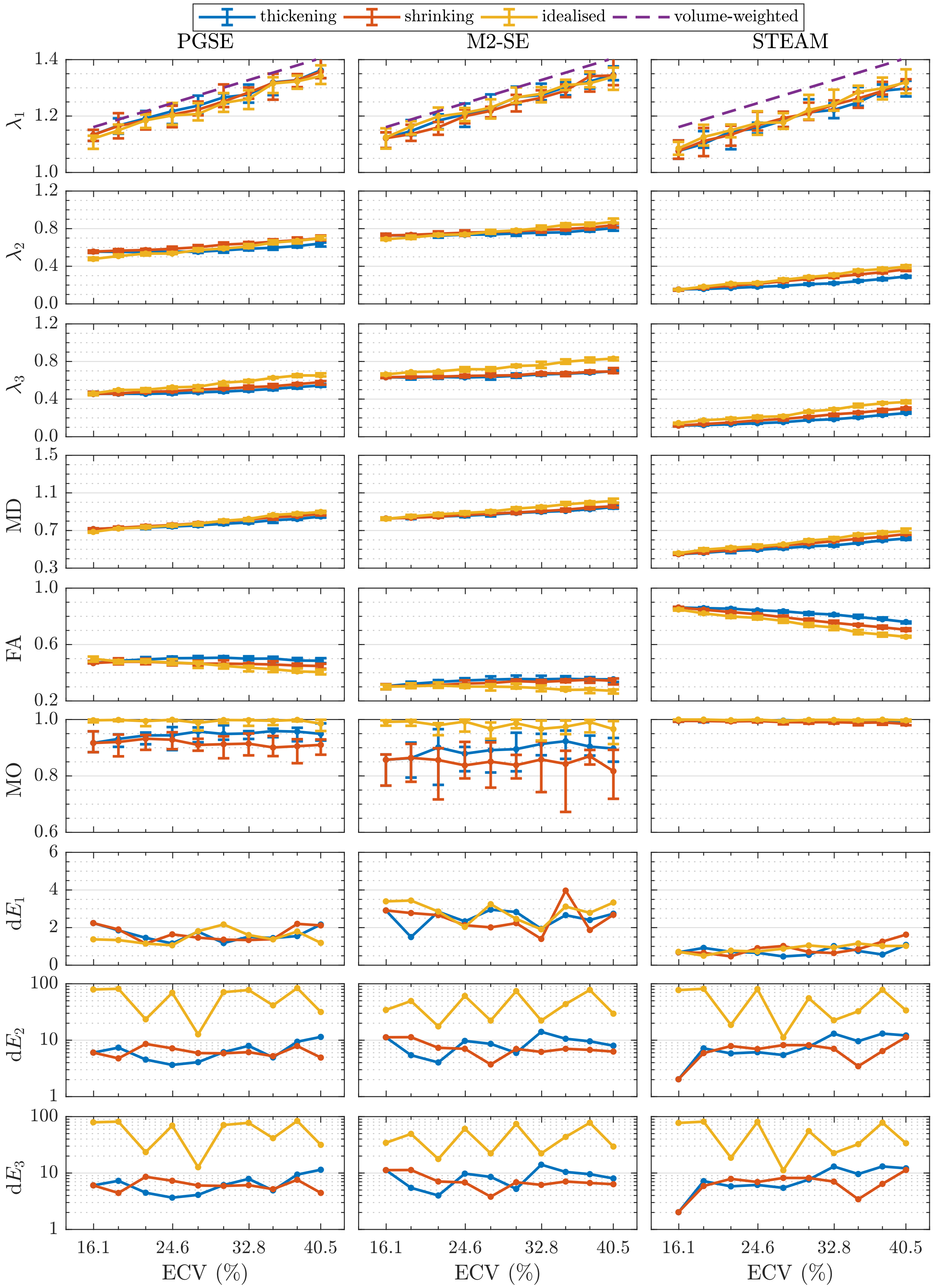

Supporting Information Figure S8: Diffusion tensor parameters as a function of extra-cellular volume fraction (ECV) and geometry type for $G_{\max} = 40\,\mathrm{mT/m}$ and diffusivity values of 1.0 and $2.0\,\mathrm{\mu m^2/ms}$, intra-cellular diffusivity (D_{IC}) and extra-cellular diffusivity (D_{EC}) respectively. The units for λ_1, λ_2, λ_3, and MD are $\mathrm{\mu m^2/ms}$ and those for $\mathrm{d}E_1$, $\mathrm{d}E_2$, and $\mathrm{d}E_3$ are deg.

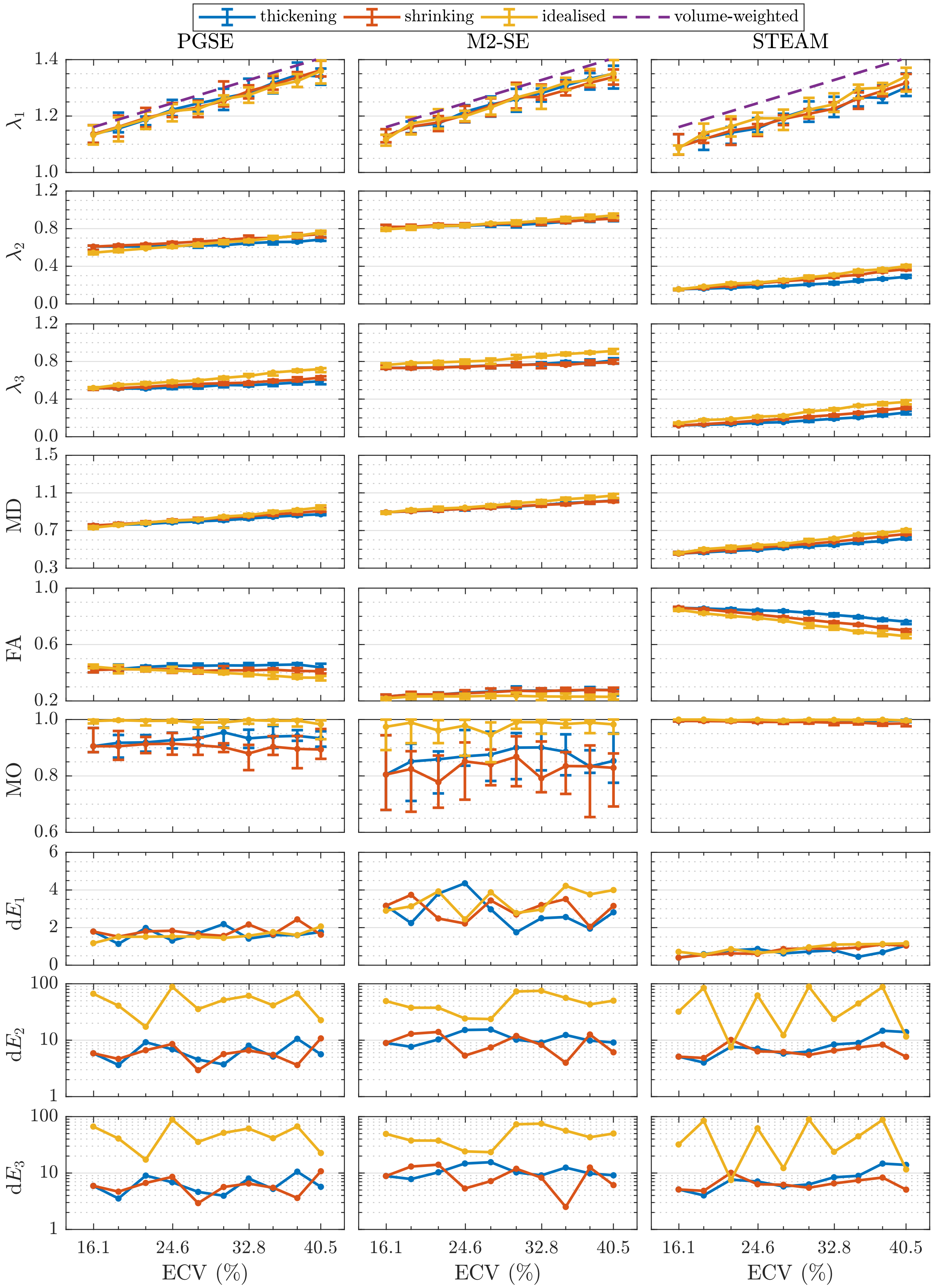

Supporting Information Figure S9: Diffusion tensor parameters as a function of extra-cellular volume fraction (ECV) and geometry type for $G_{\max} = 80\,\mathrm{mT/m}$ and diffusivity values of 1.0 and 2.0 $\mu\mathrm{m}^2$/ms, intra-cellular diffusivity (D_{IC}) and extra-cellular diffusivity (D_{EC}) respectively. The units for λ_1, λ_2, λ_3, and MD are $\mu\mathrm{m}^2$/ms and those for $\mathrm{d}E_1$, $\mathrm{d}E_2$, and $\mathrm{d}E_3$ are deg.
